# Estimated Number of Prevalent Kidney Transplant Recipients in Japan From 1964 to 2023

**DOI:** 10.3389/ti.2025.15732

**Published:** 2025-12-15

**Authors:** Hirotaka Komaba, Yosuke Nakagawa, Masahiro Koizumi, Yusuke Tomita, Michio Nakamura

**Affiliations:** 1 Division of Nephrology, Endocrinology and Metabolism, Tokai University School of Medicine, Isehara, Japan; 2 The Institute of Medical Sciences, Tokai University, Isehara, Japan; 3 Department of Transplant Surgery, Tokai University School of Medicine, Isehara, Japan

**Keywords:** dialysis, kidney failure, kidney replacement therapy, kidney translant, kidney transplantation

Dear Editors,

Kidney transplantation is one of the primary modalities of kidney replacement therapy (KRT) for patients with kidney failure. Compared with dialysis, it offers multiple advantages, including improved survival, better quality of life, and reduced healthcare costs [1, 2]. Therefore, understanding the implementation of kidney transplantation is essential for appropriate healthcare resource allocation and policy planning in each region. In Western countries, kidney transplantation is highly prevalent, with approximately 30%–50% of patients receiving KRT being transplant recipients [3, 4]. In contrast, in Japan, kidney transplantation remains a relatively limited treatment modality, primarily due to donor shortages and potential cultural factors [5–7].

The Japan Society for Transplantation, in collaboration with the Japanese Society for Clinical Renal Transplantation, has long collected detailed data at the time of transplantation, providing accurate statistics on the number of transplants and donor types [5–7]. However, long-term post-transplant follow-up is not always complete, leaving the number of kidney transplant recipients with functioning grafts (i.e., prevalent recipients) uncertain. This lack of information represents a significant limitation for evaluating the proportion of transplant recipients relative to patients on dialysis and for conducting international comparisons.

To address this, we estimated temporal trends in the number of prevalent kidney transplant recipients in Japan using summary statistics published by the Japan Society for Transplantation [5–7]. Furthermore, using data from the Japan Society for Dialysis Therapy Renal Data Registry [8], we calculated the proportion of prevalent kidney transplant recipients among all patients receiving KRT.

The annual numbers of living- and deceased-donor kidney transplants performed from 1964 to 2023, obtained from the Japan Society for Transplantation records [5–7], are summarized in Supplementary Figure S1 and Supplementary Table S1. The number of transplants was very low in the 1960s, gradually increased from the late 1970s, and has stabilized at approximately 1,500–2,000 per year since the 2010s. By 2023, a total of 47,466 transplants had been performed, comprising 39,543 living-donor and 7,923 deceased-donor transplants.

Temporal trends in the number of prevalent kidney transplant recipients, estimated based on graft survival for each era (Supplementary Figure S2), are shown in Figure 1a and Supplementary Table S2. The estimated number of prevalent recipients increased over time, reaching 27,935 living-donor recipients, 3,617 deceased-donor recipients, and a total of 31,552 recipients by 2023.

**FIGURE 1:**
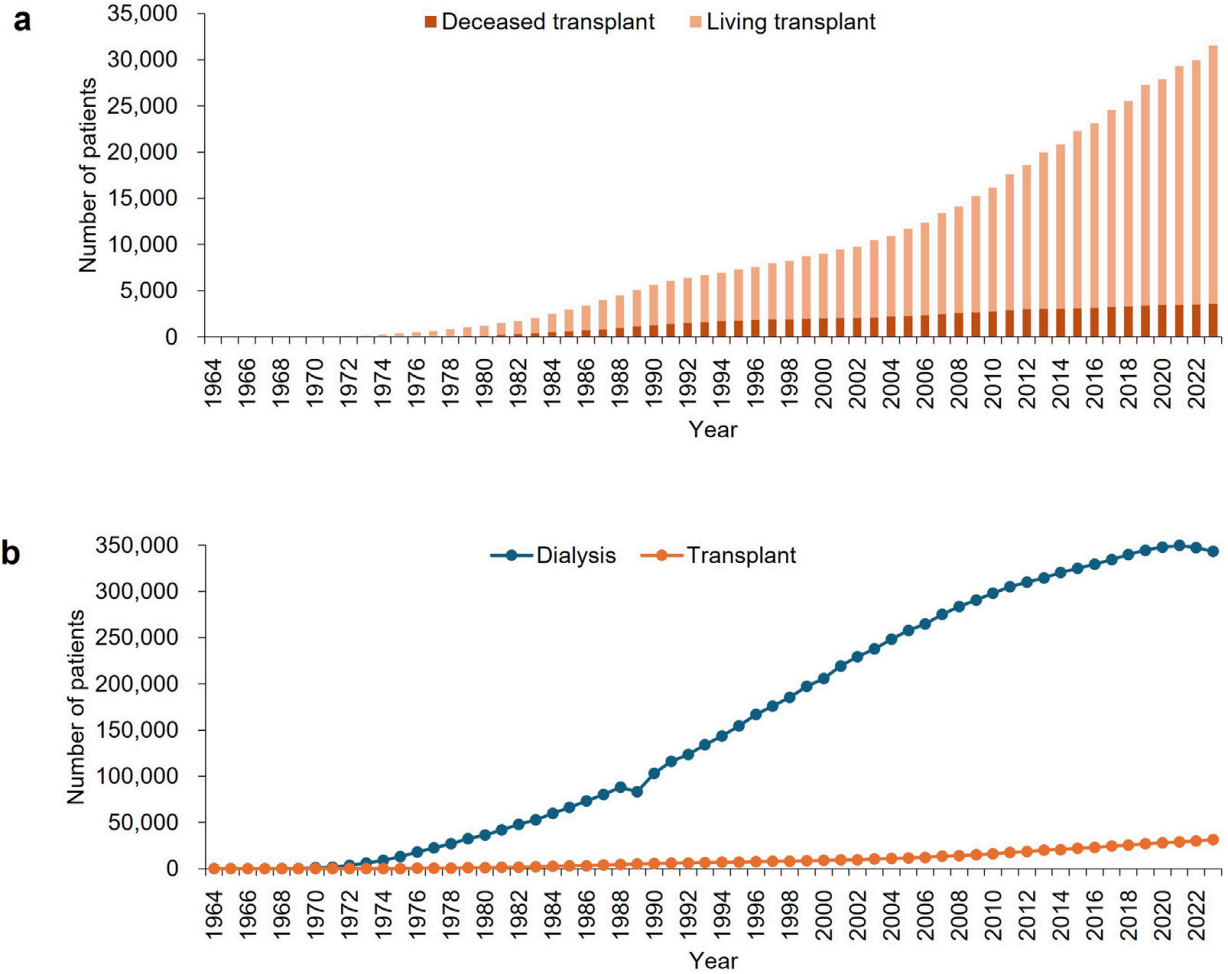
Temporal trends from 1964 to 2023 in **(a)** the estimated numbers of living- and deceased-donor prevalent kidney transplant recipients, and **(b)** the estimated total number of prevalent kidney transplant recipients, compared with the number of patients undergoing dialysis. Data on patients undergoing dialysis were obtained from the Japan Society for Dialysis Therapy Renal Data Registry [8]. Detailed numerical data are provided in Supplementary Tables S2, S3.

Temporal trends in the estimated number of prevalent kidney transplant recipients, compared with the number of patients on dialysis reported by the JSDT Renal Data Registry [8], are shown in Figure 1b and Supplementary Table S3. The proportion of these patients relative to the total population in Japan is shown in Supplementary Figure S3. The estimated proportion of prevalent kidney transplant recipients among all patients receiving KRT in Japan gradually increased over time, reaching 8.4% in 2023.

This study reports that the estimated number of prevalent kidney transplant recipients in Japan has steadily increased, surpassing 30,000 in 2023 and accounting for 8.4% of all patients receiving KRT. These data provide an important reference not only for understanding the current status of kidney transplantation in Japan, but also for enabling international comparisons and contributing to global discussions on transplantation practices.

A notable finding is that, compared with Western countries [3, 4], the proportion of prevalent kidney transplant recipients in Japan remains low, primarily due to the limited number of deceased-donor transplants. Contributing factors include delayed societal recognition of brain-dead organ donation, regulatory constraints, and challenges in obtaining family consent [9]. In addition, population aging and advances in chronic kidney disease management have led to an older demographic among patients requiring KRT [8], which may limit eligibility for kidney transplantation. Nonetheless, we observed a steady increase in prevalent recipients, likely reflecting both the gradual increase in the number of kidney transplants and the favorable long-term outcomes of kidney transplantation in Japan [5–7].

This study has several limitations. Most importantly, the estimates were derived from registry-reported transplant numbers and graft survival rates, rather than from a direct count of prevalent recipients. The graft survival rates were based on recipients with available follow-up, so outcomes of those lost to follow-up may differ. Furthermore, this secondary analysis relied entirely on summary data without access to individual-level information. Further investigation is needed to collect comprehensive patient-level data on prevalent kidney transplant recipients. The newly developed national transplant registry system, TRACER (TRAnsplant CEntral Registry), may help address these gaps.

In conclusion, kidney transplantation remains a relatively uncommon KRT modality in Japan, but the number of recipients with functioning grafts has steadily increased. Continued efforts are needed to refine these estimates and to establish a robust foundation for meaningful international comparisons.

## Data Availability

The original contributions presented in the study are included in the article/Supplementary Material, further inquiries can be directed to the corresponding author.
