# Supplementary material for: Estimated Number of Prevalent Kidney Transplant Recipients in Japan From 1964 to 2023
Source: Transpl Int. 2025 Dec 15;38:15732. doi: 10.3389/ti.2025.15732 (PMC12755172; doi:10.3389/ti.2025.15732)
Supplement: Supplementary file 1 [file Supplementaryfile1.docx]

**Supplementary Methods**

In this study, we primarily used publicly available statistics on kidney transplantation published by the Japan Society for Transplantation. The annual numbers of living- and deceased-donor kidney transplants performed from 1972 to 2023 were obtained from the Japan Society for Transplantation Fact Book 2024 (1). For transplants performed between 1969 and 1971, we referred to a 1990 report from the Japan Society for Transplantation (2). The numbers of transplants performed between 1964 and 1968 were obtained from a historical report presented at an early kidney transplantation meeting (3). Before this period, one kidney transplantation had been performed in 1956, which was excluded from the present analysis because it was an isolated case carried out under exceptional circumstances.

Long-term graft survival after kidney transplantation was estimated using era-specific data. Graft survival data for 1983–2000, 2001–2009, and 2010 onward were obtained from the Fact Book 2024 (1), while data for 1969–1982 were obtained from the Fact Book 2010 (4). Graft survival beyond the reported time points was estimated by linear interpolation between reported points and exponential extrapolation after the last observed point. Because precise data on graft survival for kidney transplants performed between 1966 and 1968 were unavailable, we assumed, based on historical records (3), a graft survival rate of 30% for the first post-transplant year, halving each subsequent year. For transplants performed between 1964 and 1965, historical records indicate that all recipients died within one month of transplantation (3), and this was accounted for in our analysis.

Using these data, we multiplied the annual numbers of living- and deceased-donor kidney transplants by era-specific graft survival rates to estimate the number of recipients with functioning grafts in each calendar year according to the year of transplantation. Based on these estimates, we calculated the total number of recipients with functioning grafts in each calendar year. We then compared these numbers with the reported numbers of patients receiving dialysis from the Japan Society for Dialysis Therapy Renal Data Registry (5). Finally, using population estimates from the Statistics Bureau, Ministry of Internal Affairs and Communications (6), we calculated the proportion of prevalent kidney transplant recipients and patients undergoing dialysis relative to the total population.

**Table S1.** Annual numbers of living- and deceased-donor kidney transplants performed from 1964 to 2023^a^

| **Year** | **Living** | **Deceased** | **Total** |  | **Year** | **Living** | **Deceased** | **Total** |
| --- | --- | --- | --- | --- | --- | --- | --- | --- |
| 1964 | 3 | 3 | 6 |  | 1994 | 399 | 199 | 598 |
| 1965 | 4 | 3 | 7 |  | 1995 | 432 | 172 | 604 |
| 1966 | 14 | 3 | 17 |  | 1996 | 453 | 186 | 639 |
| 1967 | 48 | 3 | 51 |  | 1997 | 437 | 159 | 596 |
| 1968 | 34 | 13 | 47 |  | 1998 | 510 | 149 | 659 |
| 1969 | 14 | 6 | 20 |  | 1999 | 566 | 158 | 724 |
| 1970 | 16 | 6 | 22 |  | 2000 | 603 | 146 | 749 |
| 1971 | 38 | 4 | 42 |  | 2001 | 554 | 151 | 705 |
| 1972 | 37 | 4 | 41 |  | 2002 | 637 | 122 | 759 |
| 1973 | 82 | 4 | 86 |  | 2003 | 728 | 138 | 866 |
| 1974 | 117 | 8 | 125 |  | 2004 | 731 | 173 | 904 |
| 1975 | 131 | 4 | 135 |  | 2005 | 835 | 160 | 995 |
| 1976 | 133 | 22 | 155 |  | 2006 | 941 | 197 | 1,138 |
| 1977 | 170 | 27 | 197 |  | 2007 | 1,043 | 187 | 1,230 |
| 1978 | 221 | 36 | 257 |  | 2008 | 994 | 210 | 1,204 |
| 1979 | 176 | 51 | 227 |  | 2009 | 1,122 | 189 | 1,311 |
| 1980 | 236 | 49 | 285 |  | 2010 | 1,277 | 208 | 1,485 |
| 1981 | 242 | 118 | 360 |  | 2011 | 1,386 | 212 | 1,598 |
| 1982 | 249 | 154 | 403 |  | 2012 | 1,420 | 193 | 1,613 |
| 1983 | 339 | 191 | 530 |  | 2013 | 1,438 | 155 | 1,593 |
| 1984 | 405 | 159 | 564 |  | 2014 | 1,479 | 127 | 1,606 |
| 1985 | 417 | 143 | 560 |  | 2015 | 1,503 | 167 | 1,670 |
| 1986 | 470 | 174 | 644 |  | 2016 | 1,471 | 177 | 1,648 |
| 1987 | 549 | 163 | 712 |  | 2017 | 1,544 | 198 | 1,742 |
| 1988 | 534 | 198 | 732 |  | 2018 | 1,683 | 182 | 1,865 |
| 1989 | 547 | 261 | 808 |  | 2019 | 1,827 | 230 | 2,057 |
| 1990 | 551 | 220 | 771 |  | 2020 | 1,569 | 141 | 1,710 |
| 1991 | 463 | 234 | 697 |  | 2021 | 1,649 | 125 | 1,774 |
| 1992 | 402 | 207 | 609 |  | 2022 | 1,594 | 198 | 1,792 |
| 1993 | 323 | 197 | 520 |  | 2023 | 1,753 | 248 | 2,001 |

^a^Data were obtained from the Japan Society for Transplantation records (1-3).

**Table S2.** Estimated numbers of living- and deceased-donor prevalent kidney transplant recipients, 1964–2023

| **Year** | **Living** | **Deceased** | **Total** |  | **Year** | **Living** | **Deceased** | **Total** |
| --- | --- | --- | --- | --- | --- | --- | --- | --- |
| 1964 | 3 | 3 | 6 |  | 1994 | 5,255 | 1,699 | 6,954 |
| 1965 | 4 | 3 | 7 |  | 1995 | 5,578 | 1,762 | 7,340 |
| 1966 | 14 | 3 | 17 |  | 1996 | 5,757 | 1,840 | 7,597 |
| 1967 | 52 | 4 | 56 |  | 1997 | 6,071 | 1,887 | 7,958 |
| 1968 | 51 | 14 | 65 |  | 1998 | 6,289 | 1,926 | 8,215 |
| 1969 | 32 | 11 | 43 |  | 1999 | 6,739 | 1,974 | 8,713 |
| 1970 | 37 | 11 | 48 |  | 2000 | 7,002 | 2,008 | 9,010 |
| 1971 | 67 | 11 | 78 |  | 2001 | 7,450 | 2,047 | 9,497 |
| 1972 | 94 | 12 | 106 |  | 2002 | 7,718 | 2,073 | 9,791 |
| 1973 | 169 | 13 | 182 |  | 2003 | 8,330 | 2,118 | 10,448 |
| 1974 | 265 | 18 | 283 |  | 2004 | 8,713 | 2,199 | 10,912 |
| 1975 | 374 | 18 | 392 |  | 2005 | 9,467 | 2,266 | 11,733 |
| 1976 | 470 | 37 | 507 |  | 2006 | 10,012 | 2,372 | 12,384 |
| 1977 | 618 | 52 | 670 |  | 2007 | 10,970 | 2,464 | 13,434 |
| 1978 | 778 | 73 | 851 |  | 2008 | 11,541 | 2,578 | 14,119 |
| 1979 | 942 | 103 | 1,045 |  | 2009 | 12,564 | 2,666 | 15,230 |
| 1980 | 1,071 | 124 | 1,195 |  | 2010 | 13,387 | 2,773 | 16,160 |
| 1981 | 1,273 | 212 | 1,485 |  | 2011 | 14,694 | 2,886 | 17,580 |
| 1982 | 1,408 | 301 | 1,709 |  | 2012 | 15,600 | 2,977 | 18,577 |
| 1983 | 1,666 | 406 | 2,072 |  | 2013 | 16,947 | 3,027 | 19,974 |
| 1984 | 1,988 | 512 | 2,500 |  | 2014 | 17,808 | 3,047 | 20,855 |
| 1985 | 2,327 | 602 | 2,929 |  | 2015 | 19,172 | 3,106 | 22,278 |
| 1986 | 2,694 | 722 | 3,416 |  | 2016 | 19,949 | 3,170 | 23,119 |
| 1987 | 3,156 | 822 | 3,978 |  | 2017 | 21,324 | 3,251 | 24,575 |
| 1988 | 3,545 | 955 | 4,500 |  | 2018 | 22,203 | 3,312 | 25,515 |
| 1989 | 3,976 | 1,140 | 5,116 |  | 2019 | 23,844 | 3,418 | 27,262 |
| 1990 | 4,356 | 1,267 | 5,623 |  | 2020 | 24,475 | 3,430 | 27,905 |
| 1991 | 4,702 | 1,408 | 6,110 |  | 2021 | 25,867 | 3,427 | 29,294 |
| 1992 | 4,887 | 1,513 | 6,400 |  | 2022 | 26,474 | 3,499 | 29,973 |
| 1993 | 5,101 | 1,606 | 6,707 |  | 2023 | 27,935 | 3,617 | 31,552 |

**Table S3.** Estimated numbers of prevalent kidney transplant recipients, numbers of patients undergoing dialysis, and total population in Japan, 1964–2023

| **Year** | **Transplant** | **Dialysis**^a^ | **Population**^b,c^ |  | **Year** | **Transplant** | **Dialysis**^a^ | **Population**^b,c^ |
| --- | --- | --- | --- | --- | --- | --- | --- | --- |
| 1964 | 6 | – | 97,182 |  | 1994 | 6,954 | 143,709 | 125,265 |
| 1965 | 7 | – | 98,275 |  | 1995 | 7,340 | 154,413 | 125,570 |
| 1966 | 17 | – | 99,036 |  | 1996 | 7,597 | 167,192 | 125,859 |
| 1967 | 56 | – | 100,196 |  | 1997 | 7,958 | 175,988 | 126,157 |
| 1968 | 65 | 215 | 101,331 |  | 1998 | 8,215 | 185,322 | 126,472 |
| 1969 | 43 | 301 | 102,536 |  | 1999 | 8,713 | 197,213 | 126,667 |
| 1970 | 48 | 949 | 103,720 |  | 2000 | 9,010 | 206,134 | 126,926 |
| 1971 | 78 | 1,826 | 105,145 |  | 2001 | 9,497 | 219,183 | 127,316 |
| 1972 | 106 | 3,631 | 107,595 |  | 2002 | 9,791 | 229,538 | 127,486 |
| 1973 | 182 | 6,148 | 109,104 |  | 2003 | 10,448 | 237,710 | 127,694 |
| 1974 | 283 | 9,245 | 110,573 |  | 2004 | 10,912 | 248,166 | 127,787 |
| 1975 | 392 | 13,059 | 111,940 |  | 2005 | 11,733 | 257,765 | 127,768 |
| 1976 | 507 | 18,010 | 113,094 |  | 2006 | 12,384 | 264,473 | 127,901 |
| 1977 | 670 | 22,579 | 114,165 |  | 2007 | 13,434 | 275,242 | 128,033 |
| 1978 | 851 | 27,048 | 115,190 |  | 2008 | 14,119 | 283,421 | 128,084 |
| 1979 | 1,045 | 32,331 | 116,155 |  | 2009 | 15,230 | 290,661 | 128,032 |
| 1980 | 1,195 | 36,397 | 117,060 |  | 2010 | 16,160 | 298,252 | 128,057 |
| 1981 | 1,485 | 42,223 | 117,902 |  | 2011 | 17,580 | 304,856 | 127,834 |
| 1982 | 1,709 | 47,978 | 118,728 |  | 2012 | 18,577 | 310,007 | 127,593 |
| 1983 | 2,072 | 53,017 | 119,536 |  | 2013 | 19,974 | 314,438 | 127,414 |
| 1984 | 2,500 | 59,811 | 120,305 |  | 2014 | 20,855 | 320,448 | 127,237 |
| 1985 | 2,929 | 66,310 | 121,049 |  | 2015 | 22,278 | 324,986 | 127,095 |
| 1986 | 3,416 | 73,537 | 121,660 |  | 2016 | 23,119 | 329,609 | 127,042 |
| 1987 | 3,978 | 80,553 | 122,239 |  | 2017 | 24,575 | 334,505 | 126,919 |
| 1988 | 4,500 | 88,534 | 122,745 |  | 2018 | 25,515 | 339,841 | 126,749 |
| 1989 | 5,116 | 83,221 | 123,205 |  | 2019 | 27,262 | 344,640 | 126,555 |
| 1990 | 5,623 | 103,296 | 123,611 |  | 2020 | 27,905 | 347,671 | 126,146 |
| 1991 | 6,110 | 116,303 | 124,101 |  | 2021 | 29,294 | 349,700 | 125,502 |
| 1992 | 6,400 | 123,926 | 124,567 |  | 2022 | 29,973 | 347,474 | 124,947 |
| 1993 | 6,707 | 134,298 | 124,938 |  | 2023 | 31,552 | 343,508 | 124,352 |

^a^Data on patients undergoing dialysis were obtained from the Japan Society for Dialysis Therapy Renal Data Registry (5).

^b^Total population data were obtained from the Statistics Bureau, Ministry of Internal Affairs and Communications (6).

^c^Values are expressed in units of 1,000 persons.

**Figure S1.** Temporal trends from 1964 to 2023 in the numbers of living- and deceased-donor kidney transplants performed. Data were obtained from the Japan Society for Transplantation records (1-3). Detailed numerical data are provided in Supplementary Table S1.

**Figure S2.** Era-specific graft survival of (a) living- and (b) deceased-donor prevalent kidney transplant recipients. Data were adapted from the Japan Society for Transplantation records (1,3,4).

**Figure S3.** Estimated number of prevalent kidney transplant recipients and patients undergoing dialysis per million population in Japan, 1964–2023. Data on patients undergoing dialysis were obtained from the Japan Society for Dialysis Therapy Renal Data Registry (5). Total population data were obtained from the Statistics Bureau, Ministry of Internal Affairs and Communications (6).

**Supplementary References**

1. The Japan Society for Transplantation. Fact Book 2024 on Organ Transplantation in Japan. <https://www.asas.or.jp/jst/pdf/factbook/factbook2024.pdf> (in Japanese). Date accessed: October 5, 2025
2. The Japan Society for Transplantation. Summary of clinical registration of kidney transplantation (1990). Jpn J Transplant. 1991;26(5): 494-517 (in Japanese).
3. Sonoda T, Shikata M, Miyamoto H, et al. Proceedings of the 3rd clinical meeting on kidney transplantation. Jpn J Transplant. 1970;5(2):131-184 (in Japanese).
4. The Japan Society for Transplantation. Fact Book 2010 on Organ Transplantation in Japan. <https://www.asas.or.jp/jst/pdf/fact2010.pdf> (in Japanese). Date accessed: October 5, 2025
5. Masaki T, Hanafusa N, Abe M, et al. 2023 Annual dialysis data report, JSDT renal data registry. J Jpn Soc Dial Ther. 2024;57(12):543-620 (in Japanese). <https://doi.org/10.4009/jsdt.57.543> Date accessed: October 5, 2025
6. Statistics Bureau, Ministry of Internal Affairs and Communications. Population Estimates as of October 1, 2023. https://www.stat.go.jp/data/jinsui/2023np/index.html (in Japanese). Date accessed: November 24, 2025
